# Sexual dimorphism in aipysurine sea snakes (Elapidae, Hydrophiinae)

**DOI:** 10.1098/rsos.231261

**Published:** 2023-12-13

**Authors:** R. Shine, T. G. Shine, G. P. Brown, V. Udyawer

**Affiliations:** ^1^ School of Natural Sciences, Macquarie University, Sydney, New South Wales 2109, Australia; ^2^ Australian Institute of Marine Science, Darwin, Northern Territory 0810, Australia

**Keywords:** Elapidae, Hydrophiinae, fecundity selection, mate-searching, mating system

## Abstract

The transition from terrestrial to aquatic life by hydrophiine elapid snakes modified targets of natural selection and likely affected sexual selection also. Thus, the shift to marine life also might have affected sexual dimorphism. Our measurements of 419 preserved specimens of six species of aipysurine snakes (genera *Emydocephalus* and *Aipysurus*) revealed sexual dimorphism in mean adult snout–vent length (SVL), body width relative to SVL, lengths and widths of heads and tails relative to SVL, and eye diameter relative to head length. Females averaged larger than males in all taxa, and generally were wider-bodied with shorter and wider tails and smaller eyes. For other traits, sexual dimorphism varied among species: for example, relative head length ranged from male-biased to female-biased, and head shape (width relative to length) was highly dimorphic only in *A. laevis*. The transition to marine life may have eliminated male–male combat (reducing selection for large males) and favoured visual rather than pheromone-based mate-searching (favouring larger eyes in males). Variation in head-size dimorphism may reflect intersexual niche partitioning, with different taxa following different trajectories. Repeated evolutionary transitions from terrestrial to aquatic life in snakes provide a powerful opportunity to explore selective forces on sexually dimorphic traits.

## 1. Introduction

In many species of animals, males and females differ in overall body sizes and shapes as well as in reproductive anatomy. Divergence between the sexes in morphological traits can be driven by multiple evolutionary pressures including sexual selection, fecundity selection, and intersexual niche partitioning [1,2]. Teasing apart the roles of those processes is facilitated by study systems in which organisms have simple body shapes (constraining the dimensions of dimorphism and facilitating its measurement) and also by phylogenetic shifts in habitat use. If the new habitat imposes novel constraints or opportunities, it may favour changes in morphology. For example, a shift from terrestrial to aquatic life may impose selection on locomotor ability (e.g. a hydrodynamic body shape) and sensory perception (e.g. an ability to see in water as well as in air) [3,4]. Such shifts might differentially affect the two sexes. For example, if sexual selection favours extensive mate-searching by males, morphological traits that affect locomotor speed and mate-locating ability (e.g. tail dimensions, body sizes and shapes, visual acuity) may be under divergent selection in males versus females [5].

In the present study, we describe and interpret patterns of sexual dimorphism of aipysurine sea snakes. Snakes exhibit multiple evolutionary transitions from terrestrial to marine existence, but in most lineages that transition is restricted to semi-amphibious life (laticaudines, natricines, homalopsines) or has generated few truly marine taxa (e.g. acrochordids). Only two lineages of venomous snakes, both within the family Elapidae, have spawned diverse marine radiations. Morphological and physiological differences between the *Aipysurus*–*Emydocephalus* clade and the *Hydrophis* clade attest to independent shifts to marine life, despite the close phylogenetic relationship between these two lineages [6]. Although three aipysurine taxa have been the subject of detailed field studies [7–10], the overall adaptive radiation of aipysurines (12 species) has attracted less scientific attention than has the more speciose radiation of *Hydrophis* and its allies (approx. 50 species) [11–13]. Our study thus addresses a significant knowledge gap.

## 2. Methods

### 2.1. Study species

**Figure 1.**
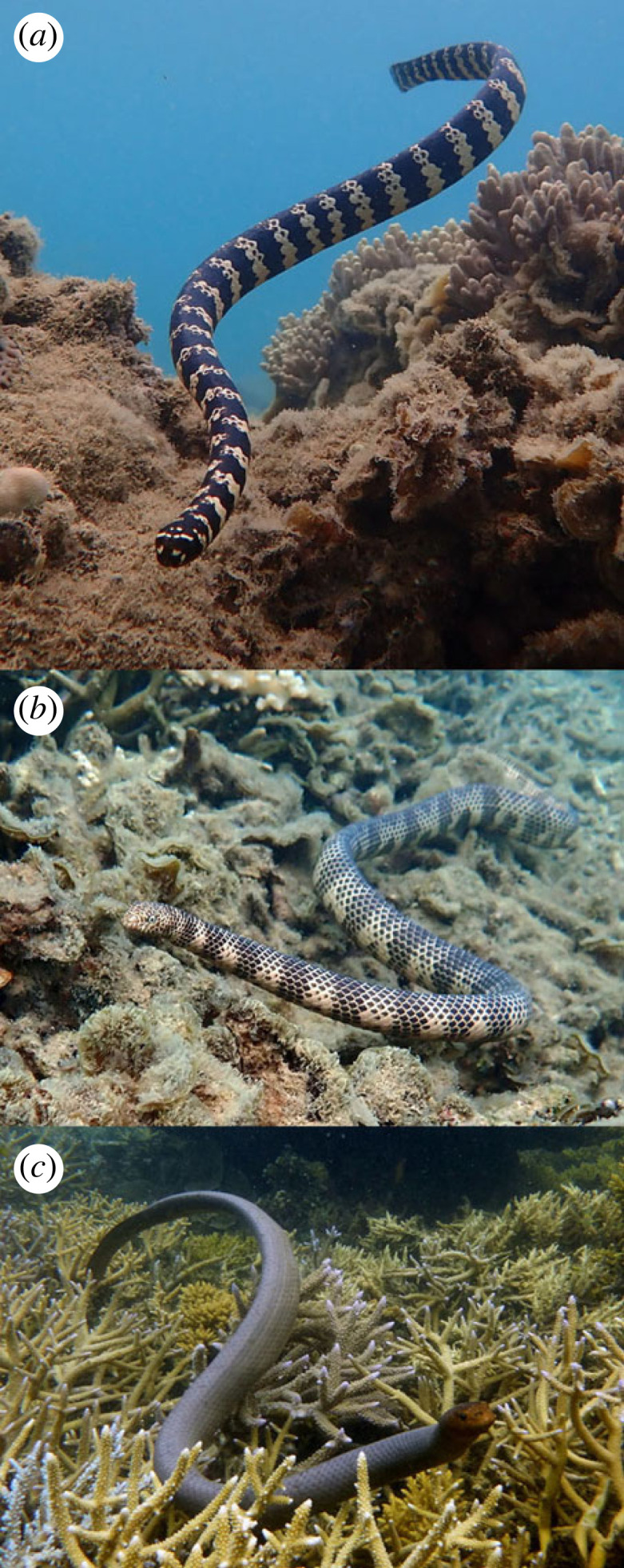
Examples of variation in body size and shape within aipysurine snakes. (*a*) *Emydocephalus annulatus* (short, stout), (*b*) *Aipysurus duboisii* (long, slender), (*c*) *Aipysurus laevis* (long, stout). All photographs by Claire Goiran.

Authorities recognize 12 species of aipysurine sea snakes, three in the genus *Emydocephalus* and nine in *Aipysurus* [14]. Most of those species are associated with coral-reef habitats in shallow-water areas from Australia to Japan [15]. Unlike the *Hydrophis* lineage, aipysurines retain enlarged ventral scales [16]. Body sizes and shapes vary considerably among species, ranging from slender (as in *A. duboisii*) to heavyset (as in *A. laevis*) (figure 1). Diets are generalized, including invertebrates such as crabs and shrimps as well as fishes and their eggs [11]. The eggs of small demersally spawning fishes are believed to be the sole or major food source for four aipysurine species (all three *Emydocephalus* plus *A. mosaicus*) [11,17].

All aipysurine species are viviparous, typically producing small litters of large offspring at less-than-annual intervals [10]. Reproduction is seasonal, with mating in winter and parturition in late summer [18]. Male sea snakes are not known to engage in combat bouts, unlike males in many terrestrial snake species [19,20]. In two aipysurine species that have been studied in detail, sexual maturity occurs at 2–3 years of age [7,10]. At least one species (*A. laevis*) exhibits sexual dichromatism, with adult males olive in dorsal coloration whereas females are steel-blue [16]. In another species (*E. annulatus*), males have a rostral spine that is used to prod the female during courtship [21]. Males of several aipysurine taxa develop rugose scales in winter (the breeding season) [22].

### 2.2. Methods for gathering data

We measured specimens of aipysurines held in collections of the Australian Museum and the Museum and Art Gallery Northern Territory. For each well-preserved specimen we recorded snout–vent length (henceforth SVL), total length and maximum width of the tail and head, and midbody girth (diameter). We used a ruler and a flexible tape to measure body dimensions, and calipers to measure maximum head width and head length (from the tip of the snout to the rear end of the quadrate-articular projection). Sex was determined by tail shape (males have thicker tailbases), verified by a midventral incision to examine gonads in ambiguous cases. Sexual maturity was evaluated based on gonadal morphology; males were classified as adult if they had thickened epididymes, and females if they had oviductal embryos or thickened oviducts.

We obtained information on 419 snakes overall, belonging to six species (table 1). Most of the other six aipysurine species are poorly represented in museum collections [17,23].

**Table 1.** Aipysurine sea snakes examined in the present study.

species	common name	total no.	no. adult males	no. adult females
*Aipysurus duboisii*	reef shallows sea snake	71	39	24
*Aipysurus fuscus*	dusky sea snake	29	15	13
*Aipysurus foliosquama*	leaf-scaled sea snake	29	20	7
*Aipysurus mosaicus*	spine-tailed sea snake	108	27	62
*Aipysurus laevis*	olive sea snake	152	58	70
*Emydocephalus annulatus*	turtle-headed sea snake	30	14	8

### 2.3. Methods for analysing data

Data conformed to assumptions of normality and variance homogeneity. To calculate relative proportions (i.e. trait values relative to those expected for an individual of that overall size), we regressed tail length against SVL, head length against SVL, head width against head length, and eye diameter against head length. In two cases we ln-transformed a variable to improve linearity in these general linear regressions (ln body width against SVL, ln tail width against tail length). Residuals from these regressions were used to quantify deviations from the average values expected for individuals of a given size.

We analysed raw data and residual scores using ANOVA with sex and species as factors, and including the interaction between those factors. Analyses of interspecific variation in mean adult body sizes only used data on adult animals; all other analyses used the entire data set. To avoid potential statistical problems with using residual scores, we also analysed relative bodily proportions using ANCOVA, whereby SVL or tail length or head length was included as a covariate, and raw data rather than residual scores were used as the dependent variables. We also looked at sexual dimorphism in absolute eye diameter, because visual acuity may depend more upon absolute eye size than eye size relative to other dimensions of the body. Interaction terms were included, but they were deleted if non-significant (*p* > 0.05) and main effects were then recalculated.

## 3. Results

### 3.1. Body sizes of adults

**Figure 2.**
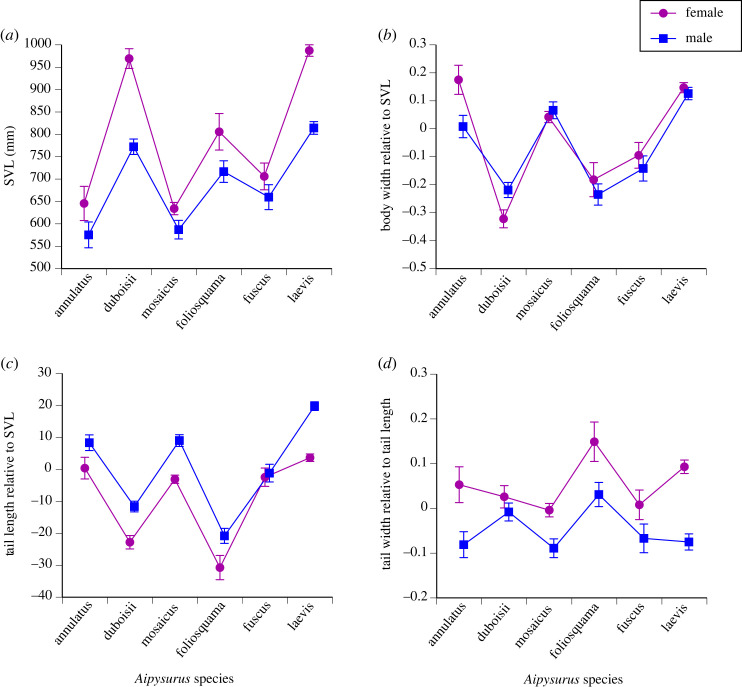
Sexual dimorphism in body sizes and proportions in aipysurine sea snakes. (*a*) Mean snout–vent lengths (SVLs) of adult males and adult females; (*b*) body shapes (ln abdominal width relative to SVL); (*c*) tail length relative to SVL; and (*d*) ln tail width relative to tail length. Dependent variables in panels (*b*) through (*d*) are based on residual scores from general linear regressions. Vertical bars show standard errors.

A two-factor ANOVA on SVLs of adult snakes yielded a strong interaction between sex and species (*F*_5,345_ = 5.65, *p* < 0.0001) because females exceeded males in size more in two taxa (*A. duboisii* and *A. laevis*) than in the other four. However, the main effect of sex (*F*_1,345_ = 48.55, *p* < 0.001) remains interpretable, because females were larger than males in all species (figure 2*a*). When each species was examined separately, adult females exceeded adult males in mean SVL in all cases (all *p* < 0.02). The two species with the most extreme size dimorphism were the largest, suggesting that this result may be influenced by allometry; but reanalysis using ln SVL rather than SVL (to examine proportional rather than absolute size differences between the sexes) also generated a significant sex × species interaction (*F*_5,345_ = 3.47, *p* < 0.005).

### 3.2. Body shape (width relative to SVL)

*Aipysurus mosaicus, A. laevis* and *E. annulatus* were more wide-bodied than were the other species (figure 2*b*). At the same SVL, females were wider-bodied than were males in *E. annulatus, A. foliosquama, A. fuscus* and *A. laevis,* but more slender-bodied in *A. duboisii*; no sex difference in relative body width was apparent in *A. mosaicus* (figure 2*b*). Thus, the interaction between sex and species was significant for relative body width (*F*_5,407_ = 2.91, *p* < 0.015). In separate ANCOVAs by species, the sexes differed significantly in relative body width in *E. annulatus, A. duboisii, A. fuscus* and *A. laevis*, but not in the other two species.

### 3.3. Tail length relative to SVL

Relative to SVL, males had longer tails than did females within all species (main effect of sex *F*_1,392_ = 50.06, *p* < 0.0001). The magnitude of that dimorphism differed among species, being minimal in *A. fuscus* compared to all other taxa (figure 2*c*; interaction sex × species *F*_5,392_ = 2.77, *p* < 0.02). In separate analyses by species, males had longer tails relative to SVL in all taxa except *A. fuscus* (all *p* < 0.0001, except *A. fuscus p* > 0.40 both in ANOVAs on residuals and in ANCOVAs with SVL as covariate and tail length as dependent variable). Tail lengths also differed among species, with *A. foliosquama* having the shortest tails relative to SVL (figure 2*c*).

### 3.4. Tail shape (width relative to length)

Males had more slender tails than did females in all species (figure 2*d*; sex effect *F*_1,369_ = 40.03, *p* < 0.0001) with less divergence in *A. duboisii* and *A. fuscus* than in the other taxa (interaction sex × species *F*_5,369_ = 22.77, *p* < 0.02). Because males also have longer tails relative to SVL (above), the interspecific divergence in relative tail width might be a secondary consequence of tail-length divergence. That is, males and females might have similar tail widths relative to SVL. To check this possibility, we calculated residual scores of ln tail width versus SVL. As predicted, the sex-based disparity in tail width relative to SVL was minor (main effect of sex *F*_1,382_ = 0.01, *p* = 0.91) and indeed, male *A. duboisii* had wider not narrower tails than did females at the same SVL (interaction sex × species *F*_5,382_ = 3.03, *p* < 0.02). In species-specific ANCOVAs, the sex difference in tail width relative to SVL was non-significant except for *A. fuscus*. Overall, then, the sexes diverged more in tail length than in tail width, relative to SVL.

### 3.5. Head length relative to SVL

**Figure 3.**
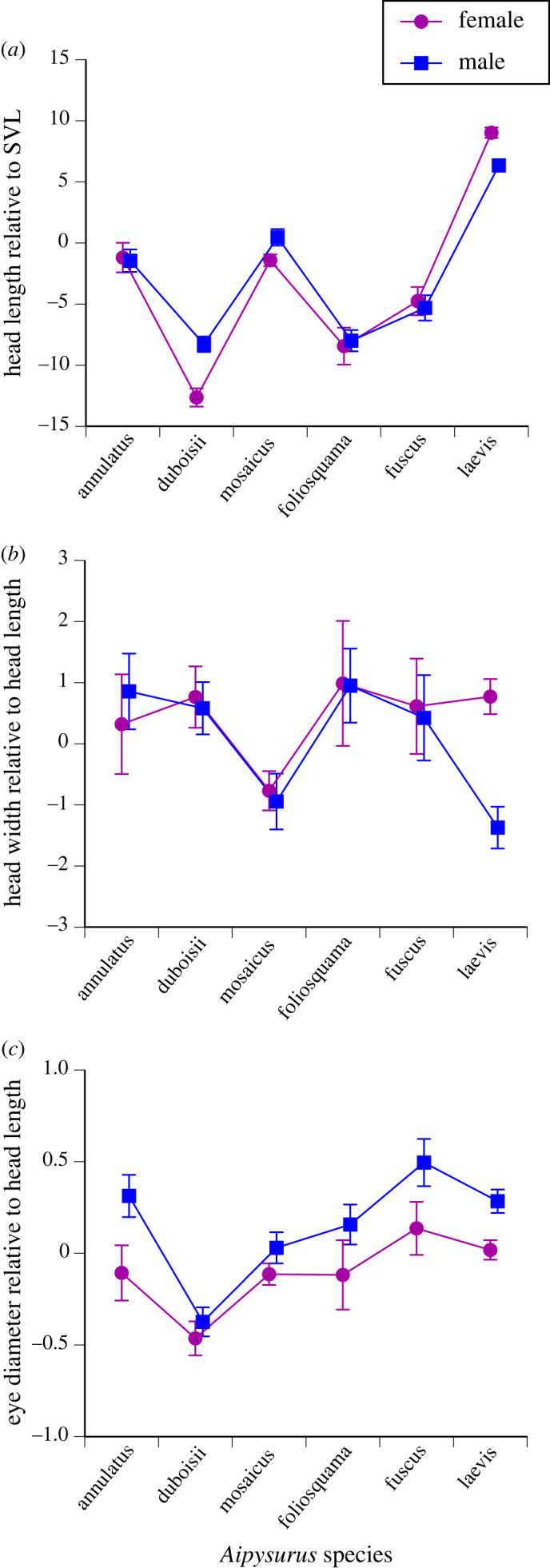
Sexual dimorphism in head and eye sizes in aipysurine sea snakes. (*a*) Head length relative to snout–vent length (SVL); (*b*) head width relative to head length; and (*c*) eye diameter relative to head length. Dependent variables in all panels are based on residual scores from general linear regressions. Vertical bars show standard errors.

On average, heads were longer relative to SVL in males than in females for *A. duboisii* and *A. mosaicus*, and smaller in males than in females for *A. laevis* (figure 3*a*; interaction sex × species *F*_5,398_ = 8.25, *p* < 0.0001). In separate ANCOVAs by species (with sex as factor, SVL as covariate and head length as dependent variable), head size increased more rapidly with increasing SVL in males than in females for *E. annulatus* (interaction sex × SVL *F*_1,26_ = 5.60, *p* < 0.03) and females had longer heads relative to SVL in *A. laevis* (sex effect *F*_1,149_ = 11.83, *p* < 0.001). Sex disparities in relative head length were marginally non-significant for *A. mosaicus*, *A. foliosquama* and *A. fuscus* (all *p* < 0.13).

### 3.6. Head shape (width relative to length)

Aipysurine species differed in head shape, with narrow heads in *A. mosaicus* of both sexes and in male *A. laevis* (figure 3*b*). The strong sex-based divergence in relative head widths of *A. laevis*, combined with lesser divergence in other taxa, yielded a significant interaction term in the ANOVA (interaction sex × species *F*_5,397_ = 2.70, *p* < 0.025). In single-species ANCOVAs, *A. laevis* was the only taxon in which the sex difference in head shape attained statistical significance (interaction sex × head length *F*_1,146_ = 8.13, *p* < 0.005).

### 3.7. Eye diameter relative to head length

Relative to head length, *A. duboisii* had smaller eyes than did the other species, and males had larger eyes than did conspecific females in all taxa (ANOVA main effect species *F*_5,398_ = 14.57, *p* < 0.0001; sex effect *F*_1,398_ = 15.69, *p* < 0.0001; interaction *F*_5,398_ = 0.73, *p* = 0.60; figure 3*c*). In single-species ANCOVAs, eye size (the dependent variable) relative to head length (the covariate) differed between the sexes (*p* < 0.05) in all species except *A. duboisii* (*p* = 0.86), *A. foliosquama* (*p* = 0.17) and *A. laevis* (*p* = 0.22).

### 3.8. Eye diameter (absolute value)

As expected, species with larger heads and bodies had larger eyes. As a result, absolute diameter of the eye averaged larger in *A. laevis* (around 5 mm) than in the other taxa (around 3 mm). Likewise, large disparities in body size between the sexes sometimes reversed the directions of sexual dimorphism apparent in eye size relative to head length. For example, females of the highly dimorphic *A. laevis* had larger eyes than did males (means of 5.32 versus 4.70 mm, *F*_1,124_ = 23.90, *p* < 0.0001) despite males having larger eyes relative to head length. In separate analyses by species, absolute eye sizes were not significantly different between adult males versus females in any species (*p* > 0.25 in all cases) except *A. laevis* (results above).

## 4. Discussion

Sex-based divergences in morphology are widespread in aipysurine sea snakes, and involve several traits. Females grew larger than males in all of the taxa that we examined, but with greater disparities in two species (*A. duboisii* and *A. laevis*) than in the other four taxa (figure 2*a*). Females also tended to be wider-bodied than males at the same SVL (except in *A. duboisii*; figure 2*b*) and to have relatively shorter tails (except in *A. fuscus*; figure 2*c*). The relationship between tail width and SVL was similar in the two sexes but tail width relative to tail length was greater in females than in males, perhaps as a consequence of tail elongation in males (figure 2*d*). Sexual dimorphism in head size relative to SVL varied among species, ranging from males-larger to females-larger (figure 3*a*). In one species (*A. laevis*), males had narrower heads than did conspecific females (figure 3*b*). Lastly, male aipysurines of at least three species had larger eyes (relative to head length) than did females (figure 3*c*). Below, we compare our results to those of previous studies, and explore evolutionary pressures that may have driven these patterns of sexual dimorphism.

Females attain larger body sizes than do conspecific males in most species of snakes, including aipysurine and hydrophiine sea snakes as well as terrestrial taxa [24,25]. Three mechanisms might favour the evolution of sex differences in mean adult body sizes:

(1) Natural selection, based on niche partitioning, if males and females consume different types or sizes of prey, or forage at different times or in different places [2,26]. Consistent with that hypothesis, field studies on aipysurines revealed sex-based differences in prey types and feeding seasons (*Emydocephalus annulatus*) [27] and in seasonal habitat use (*A. laevis*) [8]. Data are lacking for other hydrophiine sea snakes, but sea kraits (laticaudine hydrophiines) likewise show strong sex-based divergences in prey types and sizes as well as adult body sizes [26].
(2) Fecundity selection, if larger body size enhances female reproductive output [28]. This pressure should be similar among species if litter volume is constrained by maternal abdominal volume, as seems to be true in snakes [29]. That is, females of all species should obtain similar reproductive-output advantages to larger body size. However, a given reproductive burden may impose higher costs on females of some species than others. For example, a species with an unusually long pregnancy or higher vulnerability to predation might benefit from a lower relative clutch mass, which could be achieved by larger body size in females without a concomitant increase in litter mass [30]. In this scenario, larger female size would confer less benefit in a species with brief pregnancy and faced with lower threat of predation. There is no evidence of interspecific variation in such parameters, however. For example, all aipysurines appear to produce small litters of large offspring after long pregnancies [18].
(3) Sexual selection, if larger males have higher mating success because body size enhances a male's ability to defeat rival males in combat bouts [25] or in multimale wrestling matches during courtship [31,32], or to enforce copulations with females [33]. Consistent with the first of these hypotheses, male size relative to female size tends to be higher in snake species that exhibit male–male combat than in taxa that do not exhibit this behaviour [25]. Detailed field observations of two aipysurine species (*E. annulatus* and *A. laevis*) suggest that males do not fight with each other [19,34] unlike the case in the most closely related terrestrial clade (*Hemiaspis* and its allies) [6,25,35,36]. Indeed, male–male combat does not appear to have been recorded in any highly aquatic snakes [20], perhaps because tactics such as pushing a rival's head into the substrate do not work in a three-dimensional aquatic arena [25]. The apparent ubiquity of female-larger size dimorphism in sea snakes thus may reflect an evolutionary loss of male–male combat, reducing the benefit to larger body size in males.

Female aipysurines also tended to be wider-bodied than were conspecific males at the same SVL (figure 2*b*). This kind of dimorphism is widespread in other aquatic and terrestrial snakes also, and has been attributed to fecundity selection to increase the abdominal space available for a developing litter [37,38]. A wider body in males than females of one species (*A. duboisii*) is more difficult to explain. In terrestrial snakes with male–male combat, a heavyset build in males may be due to musculature that enhances success in combat bouts [37,39]. Given an apparent lack of such combat in sea snakes, however, that explanation is unlikely to apply to aipysurines.

Tail morphology differed between the sexes in our sample of aipysurines, with males having longer tails (figure 2*c*,*d*); the sex difference in relative width likely is a secondary consequence of the length dimorphism (see above). The most parsimonious explanation for longer tails in male snakes is the need to accommodate the hemipenes and retractor muscles inside the tailbase [40]. Studies on laticaudine sea kraits suggest that a male's tail length can affect both his locomotor ability and his ability to defeat rivals in courtship aggregations [5]. We note, however, that ‘tail length' *per se* may not be critical to aquatic locomotion in snakes. Swimming aipysurines flatten the entire hindbody, so the surface area available to exert propulsive force against the surrounding water may be unaffected by the exact location of the cloaca.

Even in snake species in which rival males wrestle each other for access to females, biting is rare [20]; and thus sex-based divergences in relative head size cannot be attributed to sexual selection for more powerful biting [24]. Instead, head-size dimorphism in snakes seems to reflect sex-based niche partitioning [24,41]. Longer, wider heads in female than male *E. annulatus* have been attributed to sex-based differences in foraging sites, driven by seasonal differences in the frequency of feeding and types of available prey [42]. Likewise, the sex difference in head shape in *A. laevis* (males have narrower as well as shorter heads) may reflect niche divergence; males of this species are syntopic with females on coral reefs during the breeding season, but spend the rest of the year elsewhere [8]. Diets of the two sexes were similar in reef habitat [8], but a small narrow head might confer advantages in other situations (e.g. strike hydrodynamics, or penetrating deeper into narrow burrows) [11,43].

A trend for larger eyes (relative to head length) in males rather than females (figure 3*c*) in at least three of the species we studied is intriguing, because only a small proportion of terrestrial snake taxa exhibit such divergence [44–49]. Shine *et al*. [38] reported no sex-based divergence in relative eye size in *Hydrophis major*. We envisage three plausible explanations for sex differences in relative eye size:

(1) Visual acuity depends upon absolute eye size, so that sex differences in head size (driven by niche partitioning or body-size dimorphism) might generate eyes too small or large for optimal vision unless selection adjusts eye size. That adjustment likely would result in a sex difference in eye size relative to head size [49].
(2) Sexual selection on mate-finding ability offers a plausible selective advantage for larger relative eye size in males. Unlike their terrestrial counterparts, male sea snakes cannot locate reproductive females by following substrate-deposited pheromonal trails; and instead, may rely upon visual cues to find mates until the other snake is close enough for chemosensory sampling [19,34]. The resulting difficulty in finding mates may have imposed strong selection on visual acuity in male sea snakes.
(3) Alternatively or additionally, sex differences in foraging habitats [8] may have imposed differential selection on the two sexes because of habitat-associated differences in factors such as water depth and turbidity.

Future studies could usefully extend our analyses to other snake lineages that have evolved aquatic habits. For example, there are few detailed data about sexual dimorphism in sea snakes of the *Hydrophis* group (but see [30,38,50]) or the mangrove-dwelling homalopsines and *Hydrelaps* lineages. Information on these groups, and on their terrestrial relatives, would enable a formal comparative analysis of phylogenetic changes in traits of interest. We also need detailed observations on reproductive behaviour—such as courtship, mating and male–male combat—in aquatic snakes. For logistical reasons, most of the available data for such topics are on terrestrial species [51]. Studies on captive snakes could overcome those limitations, and provide a more robust basis from which to infer the likely fitness consequences of morphological variation in aquatic snakes. More generally, phylogenetic lineages that include multiple independent adaptive transitions among habitats—between land, rivers, oceans, caves, underground burrows and the like—provide robust opportunities to clarify the ways in which novel selective regimes influence phenotypic traits differently in conspecific males and females. Multiple invasions of marine habitats by snakes offer an especially exciting model system in which to explore the evolutionary mechanisms by which organisms adapt to novel challenges, and the differential consequences of profound habitat shifts for males and females within a species.

## Data Availability

The data used in this study are open-source and publicly available at the Dryad Digital Repository at https://doi.org/10.5061/dryad.t1g1jwt82 [52].
